# Visible-light optical coherence tomography platform for the characterization of the skin barrier

**DOI:** 10.1364/BOE.494356

**Published:** 2023-07-06

**Authors:** Dmitry G. Revin, Robert A. Byers, Meng Q. Duan, Wei Li, Stephen J. Matcher

**Affiliations:** 1 Department of Electronic and Electrical Engineering, University of Sheffield, Sir Frederick Mappin Building, Sheffield, S1 3JD, UK; 2 Dermatology Research, Department of Infection and Immunity and Cardiovascular Disease, University of Sheffield, Beech Hill Road, Sheffield, S10 2RX, UK; 3 Currently with the Department of Radiology, the First Affiliated Hospital, Jinan University, No.613, Huangpu West Road, Tianhe District, Guangzhou, 510627, Guangdong, China

## Abstract

We demonstrate a free-space, trolley-mountable Fourier domain visible-light optical coherence tomography (OCT) system for studying the stratum corneum in non-palmar human skin. An axial resolution of 1 µm in tissue and at least −75 dB sensitivity have been achieved. High-quality B-scans, containing 1600 A-scans, are acquired at a rate of 39 Hz. Images from the dorsal hand, ventral wrist and ventral forearm areas are obtained, with a clearly resolved stratum corneum layer (typically 5–15 µm thick) presenting as a hypoechogenic dark layer below the bright entrance signal, similar to that found in palmar skin with traditional OCT systems. We find that the appearance of the stratum corneum layer strongly depends on its water content, becoming brighter after occlusive hydration.

## 1. Introduction

Skin barrier function is vital for overall health, as a means to prevent water loss and prevent the ingress of toxins and allergens. Some diseases are closely linked to defects in the skin barrier, for example, atopic dermatitis (AD), or eczema. This chronic inflammatory disease results in dry and cracked skin and is thought to be both genetically and environmentally influenced [1,2], affecting up to 20% of the population in developed countries [3,4]. AD is an atopic disease, meaning that it is mediated by an abnormal immune response that arises from acquired hypersensitization to external substances. It is generally believed that this hypersensitization occurs because a defective skin barrier allows allergen penetration. The severity of AD symptoms varies significantly; mild AD symptoms can be successfully treated with common emollients and/or topical corticosteroids, however, severe AD symptoms would require much stronger medical attention such as immune-suppression treatment [5]. Severe AD can degrade a patient’s quality of life very greatly. A substantial industry thus exists to develop emollients that can preserve and/or restore the integrity of the skin barrier.

It is widely believed that the skin barrier function is mainly provided by the stratum corneum (SC). SC is the top-most of 5 layers that form the epidermis and is composed of flattened, keratinized cells held together by bridging structures called corneodesmosomes. Weakening of these bridges can cause elevated shedding of cells from the SC and this can be especially pronounced in AD. The SC also influences the absorption rate of topical treatments of AD. The thickness of the SC in healthy humans is ∼5–20 µm (∼150–250 µm on the heels and palms). Since the integrity of the skin barrier can be severely compromised when affected by AD, there is potential clinical utility to be derived by developing a high axial resolution in-vivo platform that is optimized to image the SC.

Various techniques such as optical coherence tomography (OCT) [6,7], reflectance confocal microscopy [8], line-field confocal optical coherence tomography [9–11], optothermal radiometry [12], and confocal Raman spectroscopy [13] have already been applied to study human skin.

Most OCT systems operate in the near infrared (IR) range, resulting in an axial and lateral resolution typically limited to ∼5–10 µm (in tissue). In near-IR OCT systems axial resolution can be improved if a broadband laser source is used. For example, an axial resolution of ∼1.1 µm has been reported for OCT systems with very broad bandwidth (350–375 nm) laser sources, operated at ∼800 nm central wavelength [14,15]. Since the axial resolution of OCT is 0.44×λ^2^/Δλ [16] (for a Gaussian spectrum of central wavelength λ and full width half maximum (FWHM) bandwidth Δλ), OCT systems built using visible light sources [17–20] have a fundamental resolution advantage over more traditional near-IR OCT systems, achieving similar axial resolution using a smaller bandwidth. This reduces the design challenges introduced by chromatic dispersion. Also, the fundamental limits of achievable axial resolution are likely to be much better. These advantages will, however, come at the expense of reduced light penetration due to higher tissue scattering.

Reflectance confocal microscopy commercialized for in vivo skin imaging has a lateral resolution (∼1 µm) much better than that of near-IR OCT systems but can achieve a very similar axial resolution of ∼1 µm, depending on the objective numerical aperture (NA). Line-field confocal OCT is a recent innovation that has both lateral and axial resolution of ∼1 µm. A typical field of view (FOV) of ∼1.2 mm of such a technique is much smaller than that of conventional OCT systems and will hinder the observation of variations in the properties of skin that might happen on a scale of several millimeters. A small FOV also makes longitudinal measurements of the same skin area much more difficult. Image stitching also allows much larger imaging areas to be collected and this is made easier by collecting individual images with a larger FOV.

Optothermal radiometry and confocal Raman spectroscopy can only indirectly estimate the thickness of the SC but do not directly visualize the skin layers. Confocal Raman spectroscopy infers SC thickness from hydration profiles and determines these by measuring the water/lipid ratio vs depth and assuming that the lipid content does not vary with depth. Acquisition times are too slow to obtain spatially resolved measurements, in general.

Here we demonstrate a free-space, Fourier domain (FD) visible-light OCT system. The performance of this OCT system has been assessed through in vivo measurements of the SC layer in non-palmar human skin. The developed FD-OCT has an axial resolution of ∼ 1 µm in tissue, high FOV (8 mm × 8 mm) and, being Fourier domain, has the further advantage of parallelized voxel acquisition in the depth direction, improving clinical usability, and also benefiting from the well-known sensitivity advantage over time-domain OCT [21] and the high phase stability that characterizes spectrometer-based FD-OCT systems.

## 2. Visible-light OCT system

**Fig. 1.**
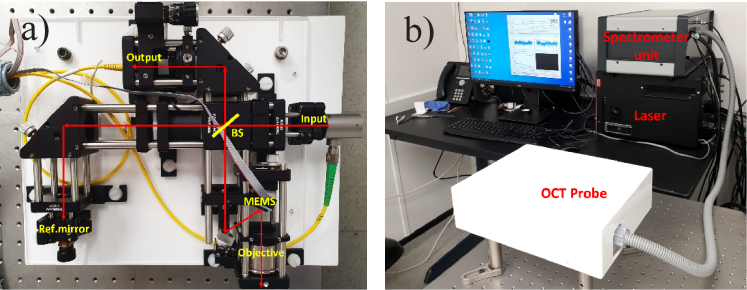
a). Free space bulk optics Michelson interferometer built using components from Thorlabs Inc., where BS houses a cube beam splitter and Ref.mirror is a mirror in the reference arm of the interferometer. b). Overall view of the visible-light FD-OCT system. The laser unit is the SuperK EXTREME EXU-6 supercontinuum laser. The spectrometer unit contains the SuperK Split unit, the spectrometer / CMOS camera and the MEMS controller. The Michelson interferometer is enclosed in the acrylic box (labelled OCT Probe). The system is controlled by a PC that is equipped with a graphical processing unit.

The visible-light FD-OCT system has been assembled using commercially available off-the-shelf components. The light source is a supercontinuum laser (SuperK EXTREME EXU-6, NKT Photonics) emitting in the wavelength range of ∼400–2400 nm with an average output power of up to 5 W and at a repetition rate of 78 MHz. The near infrared part (> ∼900 nm) of the laser emission spectrum is blocked using the SuperK Split unit. The output power of the remaining laser emission is further reduced by a partly reflecting optical plate and the laser spectrum is cropped to the visible light range (∼450–720 nm) by two bandpass filters. An additional filter (FGB37-A, Thorlabs Inc.) is used to adjust the laser spectrum to be closer to a Gaussian shape. The laser emission is directed towards a Michelson interferometer through a single mode fiber (RGB400, Corning Inc.). This fiber is coupled into a reflective collimator (RC04APC-P01, Thorlabs Inc.) to provide a ∼2.5 mm diameter free-space laser light beam inside the interferometer. The free-space bulk optics Michelson interferometer has been built based on 30-mm cage system components and optics from Thorlabs Inc. (see Fig. 1(a)). A cube beam splitter divides the incident light between the reference and sample arms with a 70:30 ratio. In the reference arm, a neutral density reflective fused silica wedged filter (1 mm thick) is used to control the reference arm light power relative to the sample arm power. The sample arm comprises an intermediate mirror which reflects the light at a ∼22-degree incident angle onto a microelectromechanical mirror (MEMS) scanner (Mirrorcle Technologies Inc.) to provide area scanning. The MEMS mirror has a diameter of 4.6 mm and its electrical drive signal contains a low pass filter with a cutoff frequency of 120 Hz, which ensures that the mirror cannot be excited at its resonant frequency of 364 Hz. A sinusoidal signal drives the fast horizontal X-axis and a sawtooth shape drives the slow vertical Y-axis in order to acquire a volume C-scan. Both waveforms are generated by a programmable controller (USB-SL MZ, Mirrocle Technologies Inc.). The light reflected from the MEMS is focused onto the tissue sample by a telecentric objective with an effective focal length of 39 mm, (LSM03-VIS, Thorlabs Inc.). Hence the beam incidence angle varies only slightly with scan position. The maximum FOV is 8 mm × 8 mm. The overall laser light attenuation is adjusted to limit the maximum laser power on the sample to ∼1 mW. The back-scattered and back-reflected light beams from the sample and reference arms interfere at the beam splitter and are coupled into a single mode output fibre (SM450, Thorlabs Inc.), which directs the light towards a spectrometer (Cobra VIS, Wasatch Photonics) with a spectral resolution of ∼ 0.1 nm. The laser spectrum is detected by a 492–698 nm CMOS 2048-pixel line array camera (OctoPlus, Teledyne e2V), attached to the spectrometer. The CMOS camera is run at a rate of 125 kHz with an acquisition time of ∼6.9 µs. Spectral data were collected by a frame grabber (Xtium-CL MX4, Teledyne DALSA, Inc.) which is synchronized with the movement of the MEMS. A dispersion-compensating fused silica block (LSM03DC-VIS, Thorlabs) and an additional wedged fused silica glass plate were inserted into the reference arm of the interferometer; these help to compensate the dispersion effects introduced by the objective and the covering window of the sample-arm MEMS. However, since the chromatic dispersion of the optics is large, it is difficult to achieve perfect dispersion compensation, due to a very limited range of available thicknesses of these wedged plates. A software package written in MATLAB (MathWorks Inc.) controls the operation of the FD-OCT system and acquires the OCT images of back-scattered intensity, which are displayed on a dB scale. The interferometer components are attached to a 6-mm thick acrylic plate and positioned inside a 310 mm × 240 mm × 85 mm acrylic enclosure (Fig. 1(b)) with a total weight of ∼3.3 kg. The whole system can easily be made portable by putting all its components on a trolley.

**Fig. 2.**
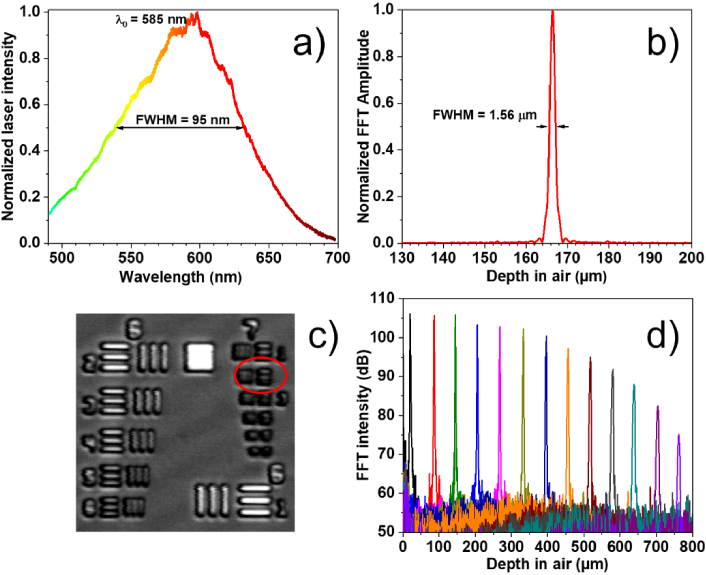
a). Spectrum of the supercontinuum laser light as detected by the Cobra VIS spectrometer & CMOS camera; b). Axial resolution of ∼1.56 µm in air, estimated by Fourier transformation of the wavenumber resampled spectral fringes obtained from a mirror in the sample arm placed at ∼167 µm away from the zero OPD; c). Transversal (lateral) resolution (better than 7 µm) estimated with a US Air Force 1951 resolution test target; d). The sensitivity roll-off of ∼−7 dB over the first 400 µm in air, estimated by translating a mirror in the sample arm.

The spectrum of the laser light incident on the sample has a shape that is close to Gaussian, with a central wavelength of λ_0_ = 585 nm and FWHM of 95 nm (Fig. 2(a)). An axial resolution of 1.56 µm in air (Fig. 2(b)) was estimated using Fourier transformation of the wavenumber- resampled spectral fringes when a mirror is placed in the sample arm at a depth of ∼167 µm away (in the direction away from the objective) from zero optical path difference (OPD). The wavenumber resampling table obtained for this mirror depth was used for the calculations of the OCT images. The depth values are calculated as a half of the optical path difference between the interferometer arms. To provide the best lateral resolution the focusing position of the objective has been adjusted to be at the depth of ∼170 µm and when obtaining the OCT images the skin surface was also being targeted to be as close as possible to this depth position. The measured axial resolution compares very well with that (1.58 µm in air) estimated as 0.44×λ_0_^2^/Δλ from the spectrum of the incident laser light. This axial resolution corresponds to ∼1 µm in the SC tissue, assuming its refractive index n = 1.55 [22]. The central wavelength and the width of the spectrum is defined and limited mainly by the commercial spectrometer used so the achievable axial resolution can be further improved if a wider part of the supercontinuum spectrum, for example, ∼ 450–750 nm, is used. Due to some remaining dispersion imbalance between the interferometer arms the axial resolution slightly worsens for higher depth values, reaching ∼ 1.9 µm in air at the depth value of ∼330 µm. To reduce this depth-dependent degradation of axial resolution, a numerical approach could potentially be used [23]. However, in our studies, we are primarily interested in the top (∼50 µm) layer of skin, whose surface is kept flat relative to zero OPD. We find that the achieved axial resolution is close enough to the transform-limited value to provide good visualization of the SC at various non-palmar skin sites.

A transversal (lateral) resolution of better than ∼7 µm in air (Fig. 2(c)) was estimated by imaging a US Air Force 1951 resolution test target (Thorlabs Inc.). This value is very similar to the theoretical transversal resolution of ∼6.9 µm calculated as 0.37×λ_0_/NA [16,24]. The maximum imaging depth in air is ∼0.85 mm (∼0.55–0.6 mm in skin tissue) and one pixel in an A-scan corresponds to ∼0.53 µm in the stratum corneum tissue. The OCT system has a roll-off of ∼−7 dB over the first 400 µm from zero OPD (Fig. 2(d)) in the direction away from the objective. This roll-off value is mainly defined by the spectrometer resolution. The sensitivity of the system, as measured from a sample arm mirror positioned close to the zero OPD, has been estimated as at least −75 dB. Such a value is not too far from the shot-noise limited sensitivity of ∼−91 dB [13] considering the relatively high (∼10 dB) intensity noise of the supercontinuum laser. B-scans containing 1600 A-scans are obtained at a rate of ∼39 Hz. The image quality can be improved by averaging several A-scans, which can attenuate background noise and speckle. However, it also reduces the image acquisition rate and hence increases subject movement artefacts. The use of A-scan averaging is thus a case of balancing these advantages and disadvantages, depending on the specific details of the imaging protocol.

## 3. OCT images of the stratum corneum

The performance of the developed visible-light OCT system has been evaluated through the imaging of the skin of one healthy volunteer. The Ethics Committee in the Electronic and Electrical Engineering department of the University of Sheffield approved the use of this system for the study of healthy volunteers (reference number 050061). Informed consent was obtained from the participant prior to imaging. OCT images were taken from various locations on the volunteer hands and forearms.

A state-of-the-art commercial dermatological near-IR OCT system (VivoSight DX, Michelson Diagnostics) was also employed to acquire OCT skin images from the same sites, for comparison. The VivoSight system uses a swept-wavelength laser source operating at a central wavelength of 1300 nm, has a depth penetration of up to 1 mm, a scanning area of 6 mm × 6 mm and a lateral and axial resolution in tissue of ∼ 7.5 µm and ∼ 5.5 µm, correspondingly.

**Fig. 3.**
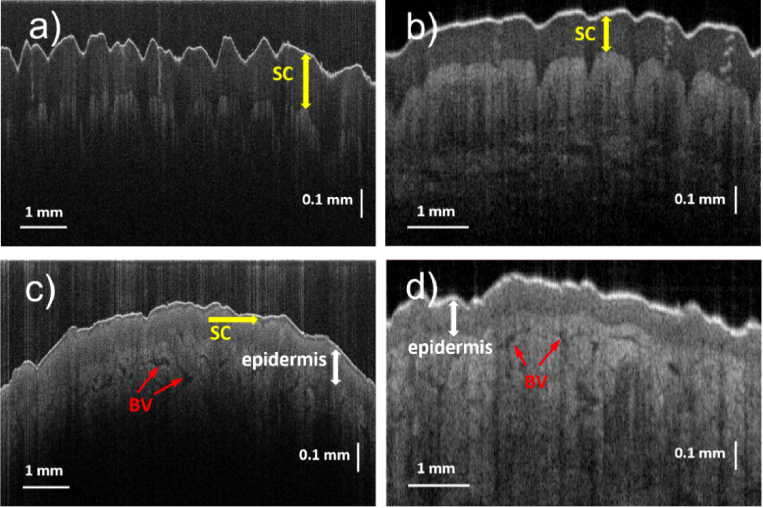
Typical B-scan images of the palm ((a), (b)) and dorsal ((c), (d)) areas of the hand of the same volunteer taken by the developed visible-light ((a), (c)) and the near-IR VivoSight ((b), (d)) OCT systems. SC – the stratum corneum layer. BV – blood vessels/capillaries. ∼130–200 µm-thick SC layers in the palm area ((a), (b)) are resolved with similar quality by both OCT techniques. However, the thin (∼12 µm) SC layer on the dorsal side is only resolved (c) by the visible-light OCT system.

Typical OCT B-scan images obtained by both the visible-light and the near-IR OCT systems from the same part of the hand (palm and dorsal sides) of a volunteer are presented in Fig. 3. The size of the images is 0.85 mm (in air) in the vertical direction and 8 mm (visible-light OCT) and 6 mm (near-IR OCT) in the horizontal direction. Each image shows a thin, bright “entrance signal” at the upper surface of the skin, due mainly to Fresnel reflection at the air/tissue interface. The stratum corneum (SC) layers are clearly visible as much darker ∼200 µm-thick layers immediately beneath the entrance signal, on the images taken at the palm area (Fig. 3(a) and (b)) using either OCT system. For the visible-light OCT system the imaging depth in the skin tissue (Fig. 3(a)) was found to be limited to just ∼300 µm due to higher scattering and absorption of the visible light compared to the near-IR light. Such an observed depth is noticeably less than that obtained with the OCT system operating in the near-IR range (Fig. 3(b)); on the other hand, it is more than enough to image the SC layer. The epidermis layer, a lighter and thicker layer beneath the SC layer, is more visible on the image obtained by the near-IR OCT system. For the palm areas (and presumably for the heels areas as well) where the thickness of the SC is expected to be several hundreds of micrometers, there is not an obvious advantage of the visible-light OCT system over the near-IR one.

However, for non-palmar areas of the skin the comparison shows the benefits of the visible-light OCT system. On the skin images taken from the dorsal side of the hand (Fig. 3(c) and (d)) the ability of the visible-light OCT system to resolve the SC layer is clear. While on the image taken by the near-IR OCT system only ∼130 µm-thick epidermis layer is visible beneath the surface layer, the visible-light OCT system can resolve the ∼12 µm-thick SC layer between the surface and the deeper epidermal layers. Other features, such as the epidermal-dermal junction and blood vessels/capillaries, present as darker formations in the dermis, are apparent on both OCT images (Fig. 3(c) and (d)). Similar to the palmar areas, the near-IR OCT system allows the light to penetrate much deeper into the dermis compared to the visible-light OCT system.

**Fig. 4.**
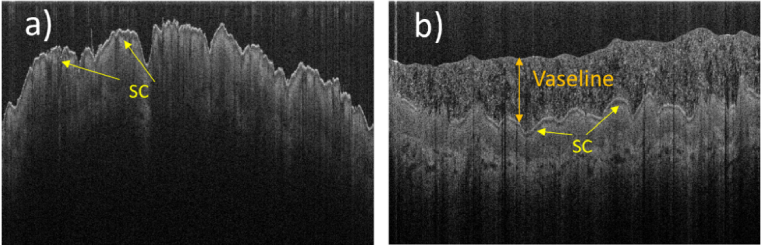
B-scan OCT images (8 mm × 0.85 mm) taken by the visible-light OCT system. (a). The ventral wrist area, where numerous thickened “spikes” directed from the SC layer towards the underlying epidermis are visible. (b). The dorsal area of the hand immediately after application of Vaseline cream. The SC layer is still clearly visible.

**Fig. 5.**
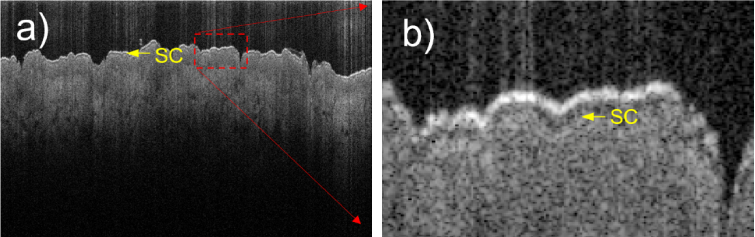
B-scan OCT images (8 mm × 0.85 mm (a) and, for clarity, a ∼1.12 mm × 0.12 mm zoomed-in detail (b)) of the ventral forearm area taken by the visible-light OCT system. The average SC thickness is ∼ 4 µm.

The observed SC thickness is ∼120–200 µm for the palm and ∼5–15 µm for non-palmar areas (Fig. 3(c), Fig. 4(a) and Fig. 5). The SC layer is not very uniform and thickness variations are often present over lateral distances of a few millimeters, highlighting the usefulness of the large (8 mm) FOV of the present OCT system. In the “transitional” areas, where the palmar type of the skin transitions into the dorsal type, such as the sides of the fingers, the palm edge and the ventral wrist, the observed SC layer becomes very uneven and has numerous thickened “spikes” directed deep down into the epidermis. Illustrative OCT image, where the SC thickness varies significantly, is presented in Fig. 4(a) for the ventral wrist area. In this area the average thickness of the SC layer is a little bit higher (∼12–16 µm, excluding the “spikes”) than in the dorsal area (∼10–14 µm) of the hand. It was found that the appearance of the SC is the best at the positions where the skin surface on OCT images is relatively flat, and the light strikes at near normal incidence. Near to micro-wrinkles, where the incident light angle is much further from normal incidence, the SC visibility becomes poorer.

OCT images have also been obtained from skin areas immediately after a thick (up to ∼200 µm) layer of Vaseline (whose chief component is petrolatum) cream was applied to them. An illustrative OCT image is presented in Fig. 4(b). For potential OCT studies of new treatments for atopic dermatitis it is important to establish how such a cream application affects the imaging of SC since petrolatum is one of the major ingredients of numerous emollients and hand creams. It was found that Vaseline reduces the bright “entrance signal” at the upper surface of the skin, has scattering properties similar to the epidermis and still allows the SC layer to be clearly identified.

Amongst the skin areas studied herein, the SC appears thinnest (∼3–5 µm) in the ventral area of the forearm, very close to the area where the majority of atopic dermatitis symptoms are found (Fig. 5). Clear delineation of the SC at this site becomes possible due to the very high axial resolution provided by our visible-light OCT system.

**Fig. 6.**
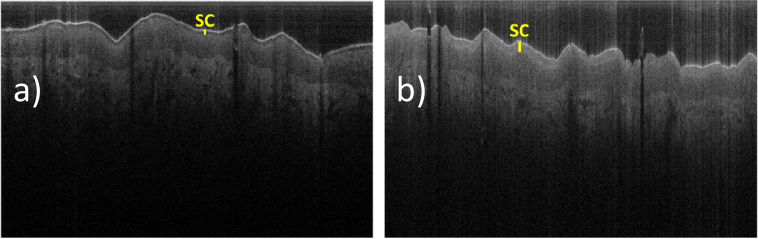
Typical B-scan OCT images (8 mm × 0.85 mm) of the same dorsal area of the hand taken by the visible-light OCT system and demonstrating the effect of an additional skin hydration. (a). The image of the skin obtained after the skin was kept at normal conditions (∼20 – 25°C, ∼25–40% humidity) for several hours. (b) The image of the skin taken after its additional hydration, when the hand of a volunteer was kept in a nitrile glove for two hours.

The light scattering properties of the top layers of the skin also depend on some external/internal factors. For example, keeping the hand of the volunteer inside a nitrile glove, for a couple of hours before the OCT imaging, results in dramatic changes in the appearance of the SC layer. We hypothesise that this effect is caused by hydration changes in the skin. The OCT images of the dorsal area of the hand, before and after wearing the glove, are presented in Fig. 6. The additional hydration possibly happens in two ways. Either the internal water from the underlying skin layers accumulated in the SC or the water from perspiration remained on the skin surface. Possibly both these factors are important, since the water evaporation from the surface of the skin was suppressed. Such a change in hydration will likely modify the back-scattering properties of the less hydrated layers of the skin, resulting in the markedly changed appearance seen here. Also, the light scattered from the surface of the skin (i.e. the entrance signal) is noticeably reduced. The back-scattering from the epidermal layers below the SC appear more uniform with depth compared with baseline (∼20–25° C, ∼25–40% of relative humidity), when these layers have a more pronounced brightness gradient (being lighter for the areas closer to the SC layer). Even more substantial changes have happened to the SC layer itself. After the hydration it appears much brighter (with the back-scattering signal increasing by ∼8–10 dB) and much thicker (∼25 µm thick vs ∼12 µm at baseline). This suggests that the back-scattering of the SC increases with the water content, among other factors.

The observed changes of the optical properties of the SC upon hydration can possibly be viewed as the inverse of the well-known “optical clearing” effect that hyperosmotic agents have on tissue [25]. Dehydration caused by hyperosmotic agents increases the extracellular refractive index and reduces the refractive index mismatch between inside and outside of a cell. This in turn reduces the scattering cross-section and, most likely, the back-scatter cross-section also. This might be the reason why the hydrated SC layer in Fig. 6(b) has a markedly higher back-scattered signal than the dehydrated one in Fig. 6(a). We believe that the observed effects are caused by hydration-related changes in both the optical properties of the top skin layers and physical swelling of these layers.

Further experiments will be conducted, to establish the detailed time evolution of skin hydration/dehydration, to compare different creams and hydration processes (e.g. immersing the hand in a water bath) and to assess, statistically, the variability across and within subjects. Other interesting experiments could include imaging SC through a greater variety of emollients, some of which may be considerably more opaque. There may then be benefits to trying to further increase the sensitivity. This may be possible by improving the light source, the spectrometer efficiency or using balanced detection to attenuate relative intensity noise.

## 4. Conclusion

A free-space, Fourier domain visible-light OCT system has been developed and its performance has been assessed through the study of the stratum corneum thickness in non-palmar human skin. An axial resolution of ∼1 µm in tissue has been achieved; this is mainly limited by the parameters of the commercial spectrometer used and could potentially be further improved. High-quality B-scans from various human non-palmar skin sites (hand, forearm) have been obtained, with a clearly resolved stratum corneum layer (down to the thickness of ∼4 µm) visible as a hypoechogenic dark layer, similar to that found in palmar skin with traditional near-IR OCT systems. The water content in the top layers of the skin greatly affects this appearance with the layer transforming into a thicker, bright layer after occlusive hydration via a nitrile glove. These observations are repeatable, in the same individual, over a time interval of several months.

The high axial resolution of the presented OCT system makes it potentially well suited to support the future development of new treatments for atopic dermatitis, where the properties of the SC layer are the therapeutic target.

## Data Availability

Data underlying the results presented in this paper are not publicly available at this time but may be obtained from the authors upon reasonable request.
